# Benzodiazepine Prescribing Within a Community Mental Health Team Setting

**DOI:** 10.1192/bjo.2024.566

**Published:** 2024-08-01

**Authors:** Azra Farooq

**Affiliations:** Pennine Care NHS Foundation Trust, Manchester, United Kingdom

## Abstract

**Aims:**

Current guidelines provide for short-term relief of symptoms using benzodiazepines, but patients, including those with complex emotional disorders often seek these medicines for longer. The current audit aims to review clinical practice in respect of benzodiazepine prescribing against national and local guidelines.

**Methods:**

Retrospective analysis of all benzodiazepines prescriptions during the study period (March–December 2021 and January–December 2023). Data was assessed against National Institute for Health and Care Excellence, British National Formulary and local Trust guidelines using a proforma and spreadsheet. The study authors separately reviewed prescribing for separate years of the study.

**Results:**

In the 2021 subsample, (9/15) 60% of patients received a benzodiazepine for less than one month. All of these patients had a psychotic disorder diagnosis. 6/15 (40%) received a benzodiazepine for more than 4 weeks, with an average duration of 5 months. Of these, only one patient had a diagnosis of a Personality Disorder. 7 patients in total (46%) were offered psychological interventions. Patients receiving benzodiazepines for more than 4 weeks were offered a tailored management plan to address their use.

In the 2023 re-audit, 10/51 (20%) patients received a benzodiazepine for greater than one month. The common indications were agitation, anxiety and crisis management. The commonest diagnoses were Personality Disorder, Post-Traumatic Stress Disorder and Schizoaffective Disorder. 4/10 (40%) patients with a Personality Disorder were prescribed a benzodiazepine for more than 4 weeks. The average duration of benzodiazepine prescribing was 11 weeks.

**Conclusion:**

Although benzodiazepines continued to be commonly used for a range of conditions, the proportion of patients not compliant with the one month, recommended duration for prescribing was reduced by half. There was a general reduction in the overall duration of prescribing but patients with a Personality Disorder continued to receive benzodiazepines for longer than recommended.

